# Impact of Vascular Anastomosis Time on Kidney Transplant Outcomes – A Systematic Review

**DOI:** 10.3389/ti.2026.15844

**Published:** 2026-03-09

**Authors:** Khaled Sayah, Henry Burt, Yizi Zheng, Taina Lee, Lawrence Yuen, Christopher Nahm, Jinna Yao, Wai Lim, Germaine Wong, Leonard Lee, Ahmer Hameed, Henry Pleass

**Affiliations:** 1 School of Medicine, Faculty of Medicine and Health, The University of Sydney, Sydney, USYD, Australia; 2 Westmead Hospital, Westmead, WMH, Australia

**Keywords:** anastomosis, second warm ischaemic time, surgical, surgical anastomosis, transplant

## Abstract

Anastomotic time (AT), also termed second warm ischaemic time (SWIT), is a potentially important intraoperative factor in kidney transplantation, yet its impact on outcomes has not been systematically synthesised. We conducted a systematic review to examine the association between AT and delayed graft function (DGF), graft survival, and patient survival. Cochrane, Embase, and Medline were searched to 21 July 2025. Nine retrospective cohort studies comprising 155,523 transplants were included. Across all donor types, longer AT was consistently associated with higher rates of DGF within individual studies. Several studies also reported poorer 1- and 5-year graft survival with prolonged AT, while findings for patient survival were equivocal. However, substantial heterogeneity across studies, including donor type, AT definitions, outcome reporting, and incomplete adjustment for key confounders, precluded formal meta-analysis. None of the included studies consistently adjusted for major determinants of graft outcomes. These findings suggest a potential link between prolonged AT and adverse graft outcomes, but high-quality prospective studies with standardised reporting and confounder adjustment are required before AT can be considered an independent determinant of transplant outcomes.

**Systematic Review Registration:**

https://www.crd.york.ac.uk/prospero/, identifier PROSPERO CRD42024549222.

## Graphical Abstract

**Figure:**
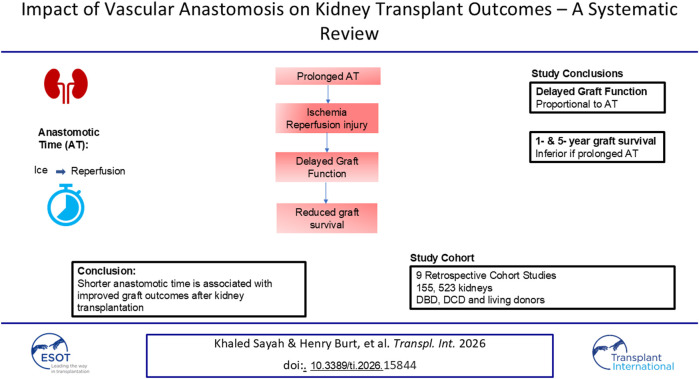

## Introduction

Kidney transplantation is the gold standard treatment for end-stage kidney disease (ESKD), offering substantial improvements in both survival and quality of life compared with dialysis [1, 2]. However, the burden of ESKD continues to grow. In the United States alone, more than 808,000 people are living with ESKD [3]. The transplant waitlist continues to expand, with 90,323 patients listed in 2024, underscoring the critical importance of optimising graft outcomes and extending the longevity of transplanted kidneys [4–6].

Outcomes after transplantation are influenced by multiple donor and recipient factors, including age, donor pathway, HLA matching, sensitisation, and organ quality [7, 8]. Among perioperative factors, cold ischaemic time (CIT) is well established as a determinant of graft function and survival [9–12].

Vascular anastomosis time (AT), also referred to as the second warm ischaemic time (SWIT), has similarly been assumed to exert an influence, but has been comparatively under-investigated, and represents the interval from removal of the kidney from ice, until reperfusion in the recipient. It has been acknowledged as a modifiable risk factor, with prolongation of this interval contributing to ischemia-reperfusion injury that drives inflammatory cascades, interstitial fibrosis and tubular atrophy (IFTA), and ultimately reduces graft viability [8, 13–18]. The clinical manifestation of this injury is delayed graft function (DGF), often requiring dialysis within the first seven days after transplantation [13–15]. Beyond early graft dysfunction, longer SWIT are also associated with longer hospital stays, increased use of renal replacement therapy, and greater reliance on diagnostic resources such as imaging and biopsy, all of which compound the burden on patients and health systems [14].

The first study to explicitly link SWIT to kidney transplant outcomes was published as recently as 2015 [8]. Since then, only a limited number of retrospective cohorts have examined this association, and no systematic review has yet synthesised the available evidence despite the critical role SWIT has on outcomes. As SWIT is a modifiable surgical factor, clarifying its effect is of direct clinical relevance. To address this, we conducted a systematic review to evaluate its association with delayed graft function, one and five-year graft survival, and patient survival in adult kidney transplant recipients.

## Materials and Methods

The systematic review was conducted in adherence with the Preferred Items for Systematic Reviews (PRISMA) checklist and was registered with PROSPERO (CRD42024549222) in 2024 [16, 17].

### Eligibility Criteria

Articles included in the systematic review were required to have been published between January 2000 – July 2025. AT (SWIT) was required to be reported with a clear description of the term, as well as type of donor graft (DCD, DBD, living donor) used in the study. Studies were required to have a sample size greater than 100, with adult recipients between 18 years or above. Transplant characteristics assessed included DGF, 1- and 5-year graft survival and patient survival. Review articles, articles with majority paediatric or elderly transplants, *en bloc* kidney transplants, multi-organ transplants, or animal studies were excluded from the systematic review.

### Literature Search and Study Selection

Supplementary Table 1 outlines the literature search strategy, developed using a combination of key words and MeSH terms. The following three databases were searched simultaneously via OVID: Cochrane databases for systematic reviews, Embase, and Medline. The final search result was performed on 21st July 2025. Results from the database search was uploaded to COVIDENCE for article screening, as outlined in Supplementary Table 1. Each included study was screened by at least two independent reviewers (KS, HB, YZ, LL), with any conflicts mediated by a third reviewer (HP).

### Data Collection

Information from each study was extracted and collated in a standardized table. The following information was documented from each article: Author(s) and study year; type of study; donor type; number of patients in the study; anastomosis time; delayed graft function; 1-year and 5-year graft survival; 1-year and 5-year patient survival. Baseline study characteristics included were Recipient BMI, Recipient Age, Donor BMI, Donor Age, Cause of death (if applicable), Donor serum creatinine, Serum urea, eGFR, and Cold Ischemic Time. This information is outlined on Tables 1, 2, 3. Data extraction was collected individually, then cross-checked by two independent reviewers before being documented on a joint spreadsheet. The terms SWIT and AT are used interchangeably throughout this manuscript.

**TABLE 1:** Baseline study characteristics.

Author and Year	Study type	Population and donor types	Recipient BMI (mean, SD) kg/m^2^	Recipient Age (mean, SD) years	Donor BMI (Mean, SD) kg/m^2^	Donor age (mean, SD) years	Cause of death	Donor Serum creatinine (mean, SD) μmoL/L	Serum Urea (μmoL/L)	eGFR (mL/min/1.73 m^2^)	Cold ischemic time (mean, SD) hours
Heylen [19]	Retrospective cohort study	13,964 (DBD (12, 806), DCD (1,158))	25 ± 0.76	54.99 ± 2.6	25 ± 2	53 ± 2.6	NR	75.14 ± 2.3	NR	NR	13.7 ± 0.9
Weissenbacher [20]	Retrospective cohort study	1,245 DBD	23.67 ± 3.74	51.02	24.81 ± 3.52 (mean)	45 ± 3	DBD: Cerebrovascular accident (587), trauma (358), other (308)	83.98	2,939	NR	14.53 ± 5.69
Marzouk [14]	Retrospective cohort study	298 (DBD (n = 282), DCD (n = 16))	78 ± 17	51 ± 13	27 ± 6 (mean)	47 ± 17	NR	67 ± 31	NR	NR	12 ± 1.3
Kukla [21]	Retrospective cohort study	554 (DBD, DCD (breakdown not available))	24.4 ± 0.09	47.6 ± 4.2	25.5 ± 0.09	42.8 ± 0.37	CVD: 24.8 (23.5–26.2), trauma 24.6 (23.4–25.7), other causes 27.8 (24.8–30.8)	NR	NR	NR	18.9 ± 0.1
Tennankore [22]	Retrospective cohort study	131, 677 (living donor (50, 587), DBD (81,120), DCD (9199)	NR	50 ± 14	NR	39 ± 16	DBD: (Anoxia: 16, 447), (CVA: 29,375), (Head trauma: 32, 754), (other: 2,514). DCD: 9,199	NR	NR	NR	11 ± 2
Mahajan [23]	Retrospective cohort study	247 (DBD, DCD (breakdown not available))	NR	53 ± 3.5	NR	51.8 ± 4.4	NR	DGF: 78 ± 12; (−) DGF: 69.3 ± 6.1	NR	1-month eGFR 36.8 ± 3.6; 1-year eGFR 40 ± 3	DGF: 840 ± 63 min; No DGF: 824 ± 59 min
Heylen [8]	Retrospective cohort study	669 (DBD)	25 ± 5	55 ± 13	NR	48 ± 15	DBD	63 ± 23	NR	3-month eGFR 47 ± 17 (n = 646); 1-year eGFR 52 ± 18 (n = 598); 2-year eGFR 52 ± 18 (n = 512); 3-year eGFR 51 ± 20 (n = 373)	15 ± 4
Cron [24]	Retrospective cohort study	6, 397 (DCD)	28 ± 1.04	55.2 ± 2.4	26.8 ± 1.1	38.9 ± 3.2	Anoxia (n = 2,724. 42.58), CVA (n = 1,046. 16.35), other/unknown (n = 313. 4.89), trauma (n = 2,314. 36.17)	70.4 ± 5.5	NR	NR	17.6 ± 1.2; adjusted odds ratio (1.02) per 1 h
Hellegering [25]	Retrospective cohort study	472 (living donor)	24	44.8	25.5	50.1	N/A	NR	2 weeks: 157, 1 month:137, 1 year:128	NR	NR

**TABLE 2:** Comparing shorter to longer anastomosis times.

Study	Short anastomosis time (30–35 min)				Long anastomosis time (>35 min)			
	AT (minutes)	Number of participants	5-year survival [95% CI]	DGF	AT	Number of participants	5-year survival [95% CI]	DGF
Heylen [19]	<35	6,083	82%	NR	Q2 = 35–44 min Q3 = 45–54 min Q4 = ≥55 min	Q2 = 4,008 Q3 = 2050 Q4 = 1823	Q2 = 81% Q3 = 78% Q4 = 75%	NR
Weissenbacher [20]	<30	NR	80.60%	NR	>30 min	NR	NR	NR
Heylen [8]	34 IQR (30–40)	659	NR	17%	NR	NR	NR	NR
Cron [24]	≤30	1731	88.20%	36.70%	Q2 = 31–38 min Q3 = 39–47 min Q4 ≥ 48 min	Q2 = 1,506 Q3 = 1,584 Q4 = 1,576	Q4 = 84.8%	Q2 = 35.2% Q3 = 40.6% Q4 = 44.0%
Marzouk [14]	30 IQR (24–45)	311	NR	18%	NR	NR	NR	NR
Kukla [21]	25.2 IQR (24.3–26.1)	555	NR	29.2%	NR	NR	NR	NR
Mahajan [23]	NR	NR	NR	NR	43 IQR (35–48)	n = 247	NR	43.3%
Tennankore [22]	Q1 = <10 Q2 = 10 - <20 Q3 = 20 - <30	Q1 = 13,456 Q2 = 3,715 Q3 = 21,627	Q1 = 78% [77%–79%] Q2 = 80% [78%–81%] Q3 = 78% [78%–79%]	NR	Q4 = 30 - <40 Q5 = 40 - <50 Q6 = 50 - <60 Q7 ≥ 60	Q3 = 38,403 Q4 = 27,058 Q5 = 10,818 Q6 = 16,600	Q4 = 75% [75%–76%] Q5 = 74% [73%–75%] Q6 = 73% [72%–74%]	NR
Hellegering [25]	29.8 min	n = 477	NR	4.40%	NR	NR	NR	NR

**TABLE 3:** Comparing short (<30 min) to longer AT (>30–35 min).

Donor Type	Study	Number of participants	DGF (%)		1-year graft survival (%)		5-year graft survival (%)		1-year patient survival (%)		5-year patient survival (%)	
			Short AT	Long AT	Short AT	Long AT	Short AT	Long AT	Short AT	Long AT	Short AT	Long AT
DBD	Heylen [8]	669	17	​	95	​	85	​	​	​	​	​
	Weissenbacher [20]	1,245	33.1	​	93	90	80.6	76.6	​	​	89.6	85.7
	Kukla [21]	554	29.2	​	​	​	​	​	​	​	​	​
DCD	Cron [24]	6,397	36	42.3	96.7	94.7	89.1	85.9	96.7	96.2	87.8	87.6
DCD + DBD	Heylen [19]	13,964	​	​	88	86	82	79.5	​	​	​	​
	Marzouk [14]	298	18.8	​	​	​	​	​	​	​	​	​
	Mahajan [23]	279	​	43.3	​	​	​	​	​	​	​	​
Living + deceased	Tennankore [22]	131,677	​	​	94	91.8	78.5	74.7	​	​	​	​
Living only	Hellegering [25]	477	4.4%	​	​	​	​	​	​	​	​	​

### Formulation of Results

Given substantial heterogenicity across included studies, including differences in donor type, AT definitions, comparator thresholds, missing covariate reporting, surgical complexity and inconsistent outcome stratification, formal meta-analysis was not undertaken following statistical consultation. Quantitative pooling of outcomes across studies was not performed, as doing so would risk biasing data or producing misleading estimates from clinically and methodologically incomparable data. Simple pooling approaches such as weighted arithmetic means were considered inappropriate for this dataset. Instead, a structured narrative synthesis was conducted. Outcomes were summarised within each study, and patterns of associated trends between AT and transplant outcomes were described across donor types. Studies were grouped by donor type (DBD, DCD, living donor and mixed cohorts) and by AT threshold where applicable. Direction and consistency of reported effects were assessed descriptively for delayed graft function, graft survival and patient survival.

### Risk of Bias Assessment

Retrospective cohort studies were assessed for risk of bias using the Newcastle-Ottawa scale (Table 4) [18]. This tool evaluates bias across multiple domains, including the representation of exposed cohort, the selection of a non-exposed cohort, ascertainment of exposure, demonstration that the outcome was not present at the initial investigation of the study, comparability of included cohorts, and assessment of the outcome with adequate follow-up time [18].

**TABLE 4:** Newcastle-Ottawa assessment.

Newcastle-Ottowa clinical assessment									
References	Representation of exposed cohort	Selection		Comparability		Outcome/Exposure		Adequecy of follow-up cohorts	Total score
		Selection of non-exposed cohort	Ascertainment of exposure	Demonstration that the outcome was not present at the start of the study	Comparability of cohort on the basis of the design or analysis controlled for confounders	Assessment of the outcome	Follow-up time was appropriate for outcomes to occur		
Heylen [19]	1	1	1	1	2	1	1	0	8 good quality
Weissenbacher [20]	1	1	1	1	2	1	1	1	9 good quality
Marzouk [14]	1	1	1	1	2	0	0	0	6 fair quality
Kukla [21]	1	1	1	1	2	1	1	1	9 good quality
Tennankore [22]	1	1	1	1	2	1	0	1	7 good quality
Mahajan [23]	1	1	1	1	2	0	1	0	7 good quality
Heylen [8]	1	1	1	1	2	1	1	0	8 good quality
Hellegering [25]	1	1	1	1	2	1	1	0	8 good quality
Cron [24]	1	1	1	1	2	1	1	0	8 good quality

### Certainty of Evidence

For each comparison, certainty of evidence was assessed using the Grading of Recommendations Assessment, Development and Evaluation (GRADE) framework [26]. Outcome chosen for certainty assessments included Delayed Graft function, 1 and 5-year Graft Survival, and 1- and 5- year Patient Survival [26]. All included studies were retrospective observational cohorts and were therefore initially rated as low-certainty evidence. Longer anastomosis time was consistently associated with higher rates of delayed graft function and poorer graft survival within individual studies, but findings for patient survival were inconsistent. Certainty was downgraded due to residual confounding, heterogenicity in anastomosis time thresholds, and incomplete adjustment for key covariates, including cold ischaemic time, graft type and surgical complexity. Overall, the certainty of evidence across outcomes was rated as low to moderate, reflecting the observational design, heterogenicity and high likelihood of residual confounding. No pooled quantitative effect estimates were generated.

## Results

**FIGURE 1:**
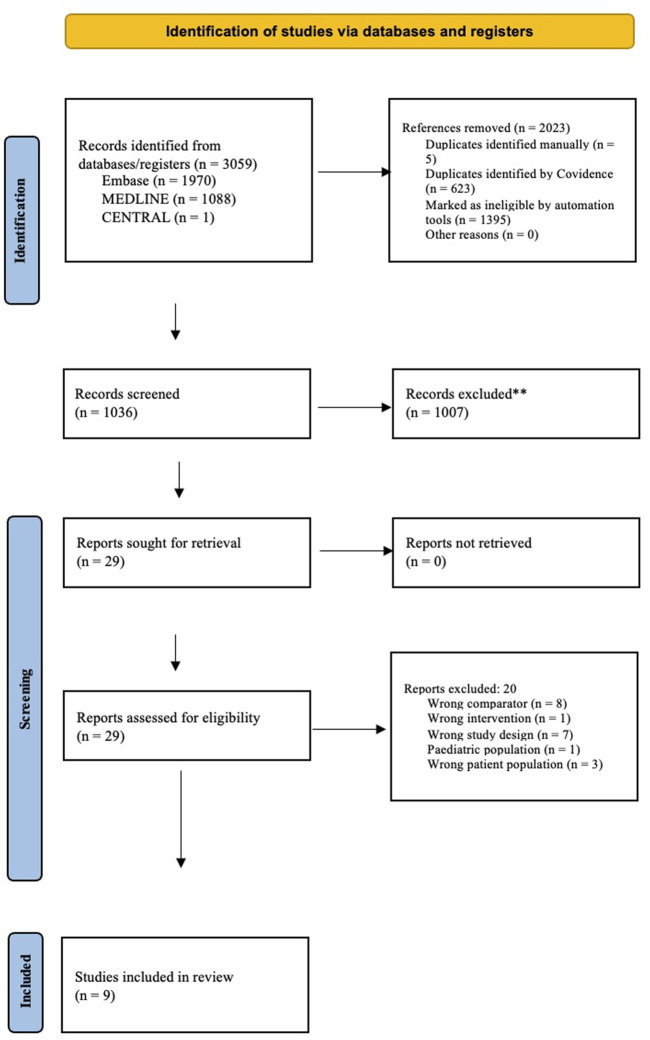
PRISMA flowchart for literature assessment.

A total of 9 retrospective cohort studies were included in the systematic review, with a total combined data spread of 155, 523 patients across two continents of North America and Europe. The PRISMA flow chart is outlined in Figure 1.

### Population Characteristics

Across the 9 retrospective cohort studies, population characteristics were sectioned into recipient and donor patient characteristics. Of the recipient population, reported BMI ranged from 23.7 kg/m^2^ to 28.0 kg/m^2^, and the median age was 51.2 IQR (48.8–55.0). With respect to the donor population, the median age was 47 IQR (40.9–70.0). Donor types were recorded as living donors (n = 51, 059), donation after circulatory death (n = 16, 770), and donation after brain death (n = 96, 122). For the deceased donor population, the cause of death was included if available in the retrospective cohort study.

### Study Characteristics

Three studies used a mixed kidney donor population of deceased donors consisting of both donation after circulatory death (DCD) and donation after brain death (DBD) [14, 19, 23]. Three studies used only DBD donors [8, 20, 21], one study limited the donor type to DCD [24], one utilized living donors [25], and the last study used a mix of both living and deceased donors [22].

### Evidence and Reporting Bias

A summary of the Newcastle-Ottawa risk of bias assessment is provided in Table 3. All studies included provided a clear description of the donor type used in their respective studies and specified the AT intervention and comparator used in their analysis. Some studies failed to provide an adequate assessment of outcome and did not provide an adequate follow-up time for long term graft outcomes to be assessed. Eight studies included maintained a good quality score ranging from 7 to 9. One of the studies included was determined as Fair quality with a rating of 6 due to the above rationale.

Overall quality of evidence was summarized in accordance with the GRADE evidence profile on Table 5 [26]. The quality of evidence was moderate for all measurable outcomes investigated. Study evidence was reduced due to all the studies included being retrospective cohort studies, and the different donor types used to assess each outcome.

**TABLE 5:** Grading of recommendations, assessment, development and evaluations (GRADE).

Outcome type	No. of studies	Risk of bias/Quality of evidence	Consistency	Directness	Precision	Publication bias	Overall effect size estimate (95% CI)	Quality of evidence
Delayed graft function (DGF)	7 (7 cohort)	Low risk of bias; observational evidence	Minimal inconsistency	Direct (0)	Narrow CI; large sample size; high precision	No important publication bias	1.10 per 10 min (1.06–1.14)	Moderate
1-year Patient Survival	3 (3 cohort)	Low risk of bias; observational evidence, however	Minimal inconsistency	Direct (0)	Narrow CI; large sample size; high precision	No evident publication bias	1.021 per minute (1.006–1.037)	Moderate
5-year Patient Survival	4 (4 cohort)	Low risk of bias; observational study	Some inconsistency	Direct (0)	Narrow CI; large sample size	No evident publication bias	3.5 (1.6–7.3)	Moderate
5-year Graft Survival	4 (4 cohort)	Low risk of bias; observational study	Minimal inconsistency	Direct (0)	Narrow CI and large sample size	No evident publication bias	1.23 (1.15–1.33)	Moderate

### Anastomosis Time Across Studies

Across the nine included studies, definitions of “short” and “long” AT varied, with thresholds ranging from <30 min to <35 min for shorter AT, and >30 min to >55 min for prolonged AT [8, 14, 21, 23–25]. Several studies reported AT quartiles rather than binary cut-offs [19, 22, 24]. In large registry-based cohorts, only a minority of recipients fell within the shortest AT category [22, 24]. Small, single centre studies reported median AT values ranging from 25 to 45 min, with wide interquartile ranges [8, 21, 23]. These findings suggest that achieving and AT <30 min is feasible in selected cases, particularly living donor transplantation, but may be uncommon in complex, deceased donor scenarios.

### Anastomosis Time and Delayed Graft Function

Seven studies analysed the relationship between vascular AT and the incidence of DGF in kidney transplantation [8, 14, 21, 23–25]. Across all donor types, longer AT were associated with higher rates of DGF (Table 2). In one study examining DBD recipients who developed DGF had significantly longer median AT compared to those without [8]. In another study examining DCD recipients, a similar effect was noted, with higher rates of DGF across increasing AT quartiles [24]. These directional associations were observed in mixed donor cohorts [19, 23], and in living donor cohorts [25], although effect sizes varied across studies. Due to heterogenicity in AT thresholds, donor populations, and outcome definitions, no pooled estimates of DGF risk were calculated.

### Anastomosis Time and Graft Survival

Five studies analysed the effect of AT on 1 and 5-year graft survival, with all studies reporting statistically significant findings, consistently demonstrating that graft survival at both time points was superior in cohorts with shorter AT [8, 19, 20, 22, 24] (Table 2). One study demonstrated a graded decline in 5-year graft survival across increasing AT quartiles [19]. One study reported inferior graft survival beyond an AT threshold of 30 min [20]. One study observed lower graft survival with prolonged AT in DCD transplantation [24]. Given differences in study design, donor populations, AT categorisations and outcome reporting, pooled graft survival estimates were not generated.

### Anastomosis Time and Patient Survival

One study with the predetermined criteria reported data on 1-year patient survival [8, 20, 24], and four reported a 5-year patient survival [8, 20, 22, 24]. For patient survival, no study found an effect of AT at 1 year, and results at 5 years were mixed. Some cohorts reported poorer survival with prolonged AT, while others showed no independent association. Taken together, the evidence suggests AT primarily influences graft-level outcomes, while its impact on patient-level survival remains uncertain [8, 20, 22, 24].

## Discussion

This review is, to our knowledge, the first systematic synthesis of vascular AT and kidney transplant outcomes across donor types. In the studies involved, prolonged AT was associated with higher rates of DGF across all donor types, and as expected the lowest incidence was seen in living donor grafts, intermediate rates in donation after brain death, and the highest rates in donation after circulatory death. In the studies involved, shorter AT, particularly under 30 min, were associated with superior graft survival, whereas both one-year and five-year graft survival declined once this interval was exceeded. Impacts on patient survival were less clear.

These findings support the interpretation that AT, DGF, and graft survival are best understood as a connected pathway rather than independent outcomes. Prolonged AT consistently increased the risk of DGF, and poorer long-term graft survival was observed in the same cohorts. Because DGF itself is a well-recognised predictor of graft loss, it is plausible that the adverse impact of prolonged AT on survival is mediated in part through its effect on early graft function. This biological plausibility is consistent with the known mechanisms of ischemia-reperfusion injury and lends coherence to the observed clinical outcomes [15].

The modifiability of vascular AT highlights its potential as a risk factor that could be improved through revised surgical techniques and guidelines. Current clinical guidelines from the Transplantation Society of Australia and New Zealand (TSANZ) do not specify an optimal vascular AT but emphasize the importance of minimising cold ischemic time [27]. Additionally, the guidelines highlight strong evidence that a short ischemic time may improve transplant outcomes, recommending that kidneys be transplanted as quickly as possible to mitigate prolonged cold ischemia [27]. However, various factors influencing vascular AT must be considered when promoting speed and precision. These include anatomical complexities (e.g., multiple renal arteries/veins, right-sided kidney grafts), recipient and donor characteristics (BMI, depth, and Age) [8, 28–30]. This emphasizes the need to recognize AT as a modifiable risk factor and incorporate its optimization into surgical planning within the transplant community and has impacts on transplant surgical training, as well as the introduction of robotic kidney transplantation more broadly.

Importantly, AT is not solely a function of surgical technique or efficiency, but also reflects surgical complexity. Prolonged AT commonly occurs in technically challenging scenarios, including ipsilateral re-transplantation, difficult iliac vessel exposure, high recipient BMI, deep iliac fossae, and the presence of calcified vessels, to name but a few [31]. In such cases, prolonged AT may be unavoidable and appropriate to ensure technical precision and haemostatic security. Accordingly, AT may act a s a surrogate marker of procedural complexity rather than an independent causal factor of poor transplant outcomes.

Cold ischaemic time is a well-established independent predictor of graft outcomes and is biologically distinct from AT [9, 11]. While AT and CIT are temporally separate, they are likely biologically synergistic. Prolonged AT may be particularly injurious in kidneys already exposed to extended cold storage, as warm re-ischemia following prolonged hypothermia may amplify ischemia reperfusion injury. This potential interaction, however, is beyond the scope of this review but remains a hypothetical, though clinically plausible mechanism, warranting prospective evaluation.

The feasibility of achieving AT below 30 min also warrants consideration. Registry based data suggests that a substantial proportion of deceased donor transplants exceed this threshold, particularly in DCD cohorts [8, 19, 20]. Short AT is most achievable in living donor cohorts, and straightforward deceased donor cases. Thresholds used across studies in this review, were arbitrary and heterogenous, and no evidence-based cut-off for “safe” AT currently exists.

These findings support viewing AT as a modifiable intra-operative factor with meaningful consequences for both early graft function and long-term graft durability. Minimisation can be encouraged through thorough preparation at the pre-operative briefing, with clear allocation of roles, readiness of instruments, and vascular exposure achieved before removal of the kidney from ice. Further, recording AT as a routine peri-operative quality measure would allow teams to monitor performance and provide constructive feedback. Workflow can also be streamlined, for example, by using a two-surgeon approach where possible and by preparing sutures or clamps in advance so that periods of non-productive time are reduced. An alternative approach would be to incorporate active methods of insulating and/or cooling the graft during anastomoses, to ameliorate the negative impacts of rapid graft rewarming and warm ischemia during this interval.

Several limitations must be recognised in this review, however. Important covariates such as cold ischaemic time, first warm ischemia, multiplicity of vessels, side of graft, recipient body mass index, machine perfusion, and centre or era effects were not consistently adjusted for. We also could not conduct formal meta-analyses and sensitivity analyses given significant differences in donor populations between different studies, variable use and reporting of technologies such as machine perfusion, and variable/inconsistent reporting of all relevant study outcomes stratified by AT thresholds and donor types.

Despite these limitations, the review has notable strengths. It is, to our knowledge, the first systematic review to focus specifically on vascular AT in kidney transplantation. Large and contemporary cohorts were included, stratified by donor type, and the overall risk of bias was low, with eight of the nine studies assessed as good quality. Taken together, this synthesis provides a structured overview of the available evidence and highlights areas where prospective research with standardised definitions and reporting would add the most value.

## Conclusion

This systematic review identified studies that found an inverse relationship between AT and graft survival. A shorter AT was associated with a superior immediate graft function, and 1-year and 5-year graft survival, across all types of donor recipients. Currently, there are no guidelines that define the significance of maintaining a short AT for optimal graft function and survival. This systematic review aims to inform the transplant community on the importance of maintaining a short AT, where possible, to provide the most optimal outcome post-operatively.

## Data Availability

The original contributions presented in the study are included in the article/Supplementary Material, further inquiries can be directed to the corresponding author.
