# Supplementary material for: Impact of Vascular Anastomosis Time on Kidney Transplant Outcomes – A Systematic Review
Source: Transpl Int. 2026 Mar 9;39:15844. doi: 10.3389/ti.2026.15844 (PMC13006796; doi:10.3389/ti.2026.15844)
Supplement: Supplementary file 1 [file Table1.docx]

**Table 1**: Literature Search Strategy

Embase <1996 to July 21 2025>

Ovid MEDLINE <1996 to 2025 July 21>

EBM Review – Cochrane Database of Systematic Reviews <2005 to July 21, 2025>

| **#** | **Keyword** | **Results** |
| --- | --- | --- |
| 1 | kidney transplantation.mp. [mp=ti, ab, hw, tn, ot, dm, mf, dv, kf, fx, dq, bt, nm, ox, px, rx, ui, sy, ux, mx, tx, kw, ct] | 214,093 |
| 2 | anastomos*.mp. [mp=ti, ab, hw, tn, ot, dm, mf, dv, kf, fx, dq, bt, nm, ox, px, rx, ui, sy, ux, mx, tx, kw, ct] | 245,808 |
| 3 | Anastomosis, Surgical/ | 84,763 |
| 4 | 1 or 2 or 3 | 455,304 |
| 5 | SWIT.mp. [mp=ti, ab, hw, tn, ot, dm, mf, dv, kf, fx, dq, bt, nm, ox, px, rx, ui, sy, ux, mx, tx, kw, ct] | 54 |
| 6 | Secondary warm isch*mic time.mp. [mp=ti, ab, hw, tn, ot, dm, mf, dv, kf, fx, dq, bt, nm, ox, px, rx, ui, sy, ux, mx, tx, kw, ct] | 3 |
| 7 | Warm isch*mia.mp. [mp=ti, ab, hw, tn, ot, dm, mf, dv, kf, fx, dq, bt, nm, ox, px, rx, ui, sy, ux, mx, tx, kw, ct] | 15,369 |
| 8 | WIT2.mp. [mp=ti, ab, hw, tn, ot, dm, mf, dv, kf, fx, dq, bt, nm, ox, px, rx, ui, sy, ux, mx, tx, kw, ct] | 27 |
| 9 | 5 or 6 or 7 or 8 | 15,430 |
| 10 | 4 and 9 | 3,059 |
| 11 | remove duplicates from 10 | 2,321 |
